# Genetic aetiology of glycaemic traits: approaches and insights

**DOI:** 10.1093/hmg/ddx293

**Published:** 2017-07-24

**Authors:** Eleanor Wheeler, Gaëlle Marenne, Inês Barroso

**Affiliations:** 1 Department of Human Genetics, Wellcome Trust Sanger Institute, Genome Campus, Hinxton, Cambridge CB10 1SA, UK; 2 Metabolic Research Laboratories, Wellcome Trust-MRC Institute of Metabolic Science, University of Cambridge, Cambridge CB2 0QQ, UK

## Abstract

Glycaemic traits such as fasting and post-challenge glucose and insulin measures, as well as glycated haemoglobin (HbA1c), are used to diagnose and monitor diabetes. These traits are risk factors for cardiovascular disease even below the diabetic threshold, and their study can additionally yield insights into the pathophysiology of type 2 diabetes. To date, a diverse set of genetic approaches have led to the discovery of over 97 loci influencing glycaemic traits. In this review, we will focus on recent advances in the genetic aetiology of glycaemic traits, and the resulting biological insights. We will provide a brief overview of results ranging from common, to low- and rare-frequency variant-trait association studies, studies leveraging the diversity across populations, and studies harnessing the power of genetic and genomic approaches to gain insights into the biological underpinnings of these traits.

## Introduction

Since their advent in 2005 (1), genome-wide association studies (GWAS) have been very successful at identifying common variant (minor allele frequency (MAF) > 5%) trait associations, with over 30,000 unique associations described to date (2). The type 2 diabetes (T2D) field has been no exception, with the number of loci robustly associated with T2D risk rising from three [*PPARG*, *KCNJ11* and *TCFL2* (3–5)] prior to the GWAS-Era, to 128 (6,7). Fasting and post-challenge glycaemic measures, and glycated haemoglobin (HbA1c), have also been the subject of intense genetic research as they are used to diagnose and monitor T2D, and are important risk factors for cardiovascular disease even within the non-diabetic range. For example, studies have found that patients diagnosed using either fasting (FG) or 2-h glucose (2hG) have distinct cardiometabolic risk (8), with 2hG being a better predictor of cardiovascular mortality than FG (9). Similarly, glycated haemoglobin (HbA1c) which reflects average glycaemia over the 2-3 month lifespan of a red blood cell, is an accepted diagnostic test for diabetes (10), but also predicts future vascular complications (11). Furthermore, insulin resistance, commonly measured using proxy phenotypes fasting insulin (FI) and insulin resistance by homeostasis model assessment [HOMA-IR (12)], is often associated with obesity or with limited peripheral adipose tissue capacity (13), and is an important risk factor for T2D. However, more sophisticated glycaemic measures such as the insulin suppression test or euglycemic clamp (considered the ‘gold standard’ estimate of peripheral insulin sensitivity) or proinsulin [adjusted for FI, equivalent to the proinsulin:insulin ratio, an indicator of beta-cell stress (14)], may, in combination with other glycaemic traits (FG, 2hG, HOMA-B and HbA1c), provide insights into diabetes pathophysiology, and possible disease stratification.

**Figure 1:**
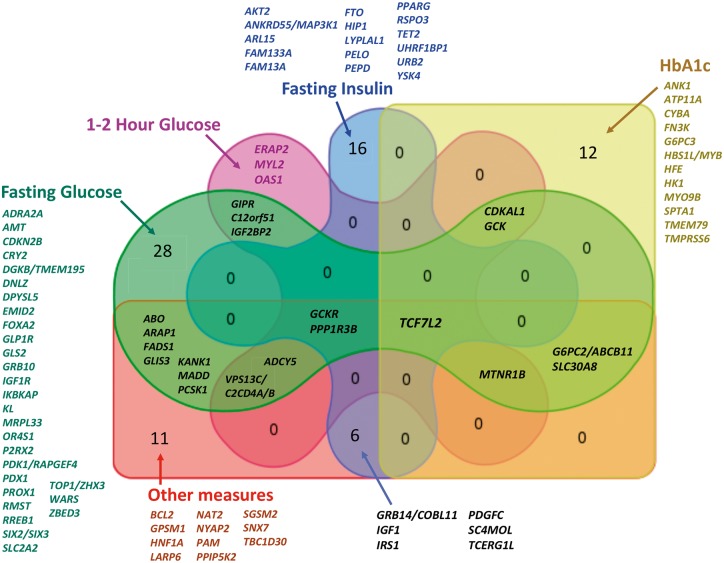
Venn diagram showing the overlap between the groups of glycaemic loci identified**.** Lists of loci (identified by the name of the closest gene to the index variant, or biologically plausible gene where known) unique to each trait, or overlapping between traits, are listed outside the diagram where that number is high, otherwise they are indicated in the figure. Loci were identified from large-scale meta-analyses with N∼108–133 K for FI and FG and N∼43–48 K for 2hrGlu, HbA1c, and HOMA-IR. Sample sizes for other glycaemic measures were much smaller, ranging from N∼16 K for ISI to just ∼1,000 participants for 1hrGlu.

The application of a series of genetic approaches to these traits have to date yielded over 97 trait-associated loci (Table 1, Fig. 1). In this review, we will focus on the progress made in recent years and will briefly describe: a) insights from common variant (MAF ≥ 5%) associations; b) results from approaches that expand the allelic frequency range to low- and rare- variant associations; c) results from diverse populations; d) early biological and functional insights and e) application of results to T2D.

**Table 1:** Loci influencing glycaemic traits

Locus	Chr	Index SNP	Refs.	Ancestry	Alleles [E/O]	Type of variant	EAF	Effect Size (SE)	P-value	Trait
*ABO*	9	rs505922	(38)	EA	C/T	Intronic	0.47	−0.038 (0.006)	3.80 × 10^−9^	Disposition index
		rs651007	(39)	EA+AA	A/G	Upstream	0.20	0.020 (0.004)	1.30 × 10^−8^	FG
*ADCY5*^b^	3	rs11708067	(80)	EA	A/G	Intronic	0.78	0.027 (0.003)	1.70 × 10^−14^	FG
		rs2877716	(89)	EA	C/T	Intronic	0.77	−0.023 (0.004)	3.6 × 10^−8^	HOMA-B
								0.09 (0.01)	4.19 × 10^−16^	2hG_adjBMI
*ADRA2A*	10	rs10885122	(80)	EA	G/T	Intergenic	0.87	0.022 (0.004)	9.70 × 10^−8^	FG
*AKT2*		rs184042322	(60)	EA	T/G	P50T	0.012	10.400 (1.990)	9.98 × 10^−10^	FI
*AMT* (RBMA6^b^)	3	rs11715915	(6)	EA	C/T	R318R	0.68	0.012 (0.002)	4.90 × 10^−8^	FG
*ANK1* (WARS^b^, NKX6-3^b^)	8	rs6474359	(90)	EA	T/C	Intronic	0.97	0.058 (0.011)	1.18 × 10^−8^	HbA1c
		rs4737009	(49)	EAA	A/G	Intronic	0.24	0.027 (0.004)	6.11 × 10^−12^	HbA1c
		rs4737009			A/G	Intronic	0.51	0.080 (0.010)	1.10 × 10^−15^	HbA1c
*ANKRD55/MAP3K1*	5	rs459193	(22)	EA	G/A	Intergenic	0.73	0.015 (0.002)	1.12 × 10^−10^	FI_adjBMI
*ARAP1* (STARD10^b^)	11	rs11603334	(22)	EA	G/A	Intronic	0.83	0.019 (0.003)	1.10 × 10^−11^	FG
			(24)				0.85	−0.093 (0.005)	3.20 × 10^−102^	Proinsulin
*ARL15*	5	rs4865796	(22)	EA	A/G	Intronic	0.67	0.015 (0.003)	2.10 × 10^−8^	FI
								0.015 (0.002)	2.20 × 10^−12^	FI_adjBMI
*ATP11A*	13	rs7998202	(90)	EA	G/A	Upstream	0.14	0.031 (0.005)	5.24 × 10^−9^	HbA1c
*BCL2*	18	rs12454712	(26)	EA	T/C	Intronic	0.58	0.050 (0.010)	1.9 × 10^−8^	ISI_adjBMI
*C12orf51* (HECTD4)	12	rs2074356	(33)	EAA	T/NR	Intronic	NR	−0.061 (0.008)	6.03 × 10^−14^	FG
								−0.321 (0.039)	1.04 × 10^−16^	1hGlu
								−0.165 (0.028)	5.91 × 10^−09^	2hG
*CDKAL1*	6	rs9368222	(22)	EA	A/C	Intronic	0.28	0.014 (0.002)	1.00 × 10^−9^	FG
		rs7747752	(91)	EAA	C/G	Intronic	0.48	0.016 (0.002)	4.54 × 10^−11^	FG_adjBMI
		rs7772603	(49)	EAA	C/T	Intronic	0.42	−0.310 (NR)	1.50 × 10^−8^	HbA1c
		rs9348440	(33)	EAA	A/NR	Intronic	NR	0.060 (0.010)	3.50 × 10^−8^	HbA1c
								0.246 (0.028)	3.13 × 10^−19^	1hGlu
*CDKN2B*	9	rs10811661	(22)	EA	T/C	Upstream	0.82	0.024 (0.003)	5.60 × 10^−18^	FG
								0.023 (0.003)	5.12 × 10^−15^	FG_adjBMI
*CRY2*	11	rs11605924	(80)	EA	A/C	Intronic	0.49	0.015 (0.003)	8.10 × 10^−8^	FG
*CYBA*	16	rs9933309	(49)	EAA	C/T	Intronic	0.63	0.070 (0.010)	1.10 × 10^−8^	HbA1c
*DGKB*^b^/*TMEM195*	7	rs2191349	(80)	EA	T/G	Intergenic	0.52	0.030 (0.003)	5.30 × 10^−29^	FG
*DNLZ*	9	rs3829109	(22)	EA	G/A	Intronic	0.71	0.017 (0.003)	1.10 × 10^−10^	FG
*DPYSL5*	2	rs1371614	(92)	EA	T/C	Intronic	0.25	0.020 (0.006)	1.33 × 10^−12^	FG_adjBMI
								0.015 (0.006)	0.00021	FG_BMI30
*EMID2*	7	rs6947345	(93)	EA	C/T	Intronic	0.98	0.162 (0.029)	3.80 × 10^−8^	FG
*ERAP2*	5	rs1019503	(22)	EA	A/G	3’UTR	0.48	0.063 (0.011)	8.90 × 10^−9^	2hG
*FADS1*^b^	11	rs174550	(80)	EA	T/C	Intronic	0.64	0.017 (0.003)	8.30 × 10^−9^	FG
								−0.020 (0.003)	5.30 × 10^−10^	HOMA-B
*FAM133A*	X	rs213676	(46)	AA	C/G	Intergenic	0.98	0.147 (NR)	2.37 × 10^−8^	FI_adjBMI
*FAM13A*	4	rs3822072	(22)	EA	A/G	Intronic	0.48	0.012 (0.002)	1.90 × 10^−8^	FI_adjBMI
*FN3K*	17	rs1046896	(90)	EA	T/C	Upstream	0.31	0.035 (0.003)	1.57 × 10^−26^	HbA1c
*FOXA2*^b^	20	rs6048205	(92)	EA	A/G	Downstream	0.95	0.023 (0.012)	0.0014	FG_BMI30
		rs6113722	(22)	EA	G/A	Downstream	0.96	0.035 (0.005	2.50 × 10^−11^	FG
*FTO*	16	rs1421085	(22)	EA	C/T	Intronic	0.42	0.020 (0.003)	1.90 × 10^−15^	FI
*G6PC2*^b*/ABCB11*^	2	rs560887	(80)	EA	C/T	Intronic	0.70	0.075 (0.003)	8.50 × 10^−122^	FG
		rs552976	(90)	EA	G/A	Intronic	0.62	−0.042 (0.004)	7.60 × 10^−29^	HOMA-B
		rs138726309	(40)	EA	T/C	H177Y	0.01	0.032 (0.004)	1.00 × 10^−17^	HbA1c
		rs492594	(49)	EAA	C/G	V219L	0.48	0.047 (0.003)	8.16 × 10^−18^	HbA1c
		rs3755157			T/C	Intronic	0.34	−0.102 (-0.02)	3.10 × 10^−8^	FG_adjBMI
								0.02 (-0.004)	6.00 × 10^−9^	FG_adjBMI
								0.07 (0.01)	2.80 × 10^−11^	HbA1c
*G6PC3*	17	rs12602486	(50)	Malay	G/T	Downstream	0.03	−0.362 (0.035)	1.00 × 10^−4^	HbA1c
*GCK*^b^	7	rs4607517	(80)	EA	A/G	Upstream	0.16	0.062 (0.004)	1.20 × 10^−44^	FG
		rs6975024	(22)	EA	C/T	Upstream	0.15	0.103 (0.016)	5.20 × 10^−11^	2hG
		rs1799884	(90)	EA	C/T	Upstream	0.18	0.038 (0.004)	1.45 × 10^−20^	HbA1c
		rs1799884	(33)	EAA	A/NR	Intronic	NR	0.063 (0.007)	4.53 × 10^−18^	FG
								0.208 (0.035)	2.82 × 10^−9^	1hGlu
								0.162 (0.026)	2.59 × 10^−10^	2hG
*GCKR*^b^	2	rs780094	(80)	EA	C/T	Intronic	0.62	0.029 (0.003)	1.70 × 10^−24^	FG
		rs1260326	(89)		T/C	L446P	0.42	0.032 (0.004)	3.60 × 10^−19^	FI
								0.035 (0.004)	5.0 × 10^−20^	HOMA-IR
								0.100 (0.01)	7.05 × 10^−11^	2hG_adjBMI
*GIPR*	19	rs2302593	(22)	EA	C/G	Intronic	0.50	0.014 (0.002)	9.30 × 10^−10^	FG
		rs10423928	(89)	EA	A/T	Downstream	0.18	0.09 (0.01)	1.98 × 10^−15^	2hG_adjBMI
*GLIS3*	9	rs7034200	(80)	EA	A/C	Intronic	0.49	0.018 (0.003)	1.20 × 10^−9^	FG
								−0.020 (0.004)	8.9 × 10^−9^	HOMA-B
*GLP1R*	6	rs10305492	(39)	EA+AA	A/G	A316T	0.01	−0.09 (0.013)	3.40 × 10^−12^	FG
			(40)	EA			0.02	−0.073 (0.015)	4.60 × 10^−7^	FG_adjBMI
*GLS2*	12	rs2657879	(22)	EA	G/A	L581P	0.18	0.016 (0.003)	3.90 × 10^−8^	FG_adjBMI
*GPSM1*	9	rs60980157	(38)	EA	T/C	S391L	0.30	0.072 (0.013)	1.4 × 10^−8^	Insulinogenic index
*GRB10*	7	rs6943153	(22)	EA	T/C	Intronic	0.34	0.015 (0.002)	1.60 × 10^−12^	FG
*GRB14/COBL11*	2	rs10195252	(22)	EA	T/C	Upstream	0.59	0.016 (0.003)	4.90 × 10^−7^	FI
		rs7607980	(92)	EA	T/C	N939D	0.60	0.017 (0.002)	1.30 × 10^−10^	FI_adjBMI
		rs7607980	(40)	EA	T/C	N939D	0.86	0.039 (0.008)^c^	4.90 × 10^−7^	FI_BMI30
							0.89	−0.071 (0.030)	3.00 × 10^−13^	HOMA-IR
								0.030 (0.006)	6.70 × 10^−8^	FI_adjBMI
*HBS1L/MYB*	6	rs9399137	(49)	EAA	T/C	Intronic	0.69	0.07 (0.01)	8.50 × 10^−15^	HbA1c
*HFE*	6	rs1800562	(90)	EA	G/A	C282Y	0.94	0.063 (0.007)	2.59 × 10^−20^	HbA1c
*HIP1*	7	rs1167800	(22)		A/G	Intronic	0.54	0.016 (0.003)	2.60 × 10^−9^	FI
*HK1*	10	rs16926246	(90)	EA	C/T	Intronic	0.90	0.089 (0.004)	3.11 × 10^−54^	HbA1c
*HNF1A*	12	rs2650000	(38)	EA	A/C	Intergenic	0.46	−0.076 (0.012)	5.0 × 10^−10^	Insulinogenic index
*IGF1*	12	rs35767	(80)	EA	G/A	Upstream	0.85	0.010 (0.006)	0.10	FI
		rs35747	(92)	EA	A/G		0.82	0.013 (0.006)	0.04	HOMA-IR
								0.021 (0.004)	8.85 × 10^−10^	FI_adjBMI
*IGF1R*	15	rs2018860	(48)	EAA	A/T	Intronic	0.46	0.031 (0.006)	2.99 × 10^−8^	FG_adjBMI
*IGF2BP2*	3	rs7651090	(22)	EA	G/A	Intronic	0.31	0.013 (0.002)	1.75 × 10^−8^	FG
							0.30	0.064 (0.012)	4.50 × 10^−8^	2hG_adjBMI
*IKBKAP*	9	rs16913693	(22)	EA	T/G	Intronic	0.97	0.043 (0.007)	3.50 × 10^−11^	FG
*IRS1*	2	rs2943634	(92)	EA	C/A	Downstream	0.66	0.021 (0.010)	0.0036	FI_BMI30
		rs2972143	(22)	EA	G/A	Downstream	0.62	−0.015 (0.018)	2.00 × 10^−10^	HOMA-IR
		rs2943645	(22)	EA	T/C	Downstream	0.63	0.014 (0.003)	3.20 × 10^−8^	FI
								0.019 (0.002)	2.30 × 10^−19^	FI_adjBMI
*KANK1*	9	rs3824420	(38)	EA	A/G	R667H	0.03	0.107 (0.018)	1.6 × 10^−9^	Proinsulin AUC_0-30_
		rs10815355	(48)	EAA	T/G	Intronic	0.22	0.045 (0.007)	1.26 × 10^−9^	FG_adjBMI
*KL*	13	rs576674	(22)	EA	G/A	Upstream	0.15	0.017 (0.003)	2.30 × 10^−8^	FG
*LARP6*	15	rs1549318	(24)	EA	T/C	Downstream	0.61	0.019 (0.005)	2.4 × 10^−10^	Proinsulin
*LYPLAL1*	1	rs2820436	(22)	EA	C/A	Downstream Downstream	0.67	0.015 (0.003)	4.40 × 10^−9^	FI
		rs4846565	(22)	EA	G/A	Downstream	0.67	0.013 (0.002)	1.80 × 10^−9^	FI_adjBMI
		rs2785980	(92)	EA	T/C		0.67	0.018 (0.010)	0.097	FI_BMI30
*MADD*^b^ (ACP2^b^)	11	rs7944584	(80)	EA	A/T	Intronic	0.75	0.021 (0.003)	5.10 × 10^−11^	FG
		rs10501320	(24)	EA	G/C	Intronic	0.72	0.081 (0.006)	1.1 × 10^−88^	Proinsulin
		rs10838687	(24)	EA	T/G	Intronic	0.80	0.025 (0.005)	6.9 × 10^−12^	Proinsulin
		rs35233100	(38)	EA	T/C	R766X	0.04	−0.100 (0.013)^a^	7.6 × 10^−15^	Fasting proinsulin
*MRPL33*	2	rs3736594	(92)	EA	A/C	Intronic	0.28	0.022 (0.003)	5.22 × 10^−16^	FG_adjBMI
*MTNR1B*^b^	11	rs10830963	(80)	EA	G/C	Intronic	0.30	0.067 (0.003)	1.10 × 10^−102^	FG
		rs1387153	(90)	EA	T/C	Upstream	0.27	−0.034 (0.004)	1.1 × 10^−22^	HOMA-B
		rs10830962	(33)	EAA	C/NR	Upstream	NR	0.024 (0.004)	3.00 × 10^−9^	HbA1c
								0.028 (0.004)	3.96 × 10^−11^	HbA1c
								0.041 (0.006)	4.84 × 10^−13^	FG
								0.191 (0.027)	3.24 × 10^−12^	1hGlu
*MYL2*	12	rs12229654	(33)	EAA	G/NR	Intergenic	NR	−0.277 (0.039)	8.83 × 10^−13^	1hGlu
*MYO9B*	19	rs11667918	(49)	EAA	C/T	Intronic	0.62	0.060 (0.010)	9.00 × 10^−12^	HbA1c
*NAT2*^c^	8	rs1208	(25)	EA	A/G	K268R	0.57	−0.130 (0.03)	9.81 × 10^−7^	Insulin sensitivity
*NYAP2*	2	rs13422522	(26)	EA	C/G	Intergenic	0.77	−0.060 (0.010)	1.2 × 10^−11^	ISI_adjBMI
*OAS1*	12	rs11066453	(33)	EAA	G/NR	Intronic	NR	−0.242 (0.041)	4.54 × 10^−09^	1hGlu
*OR4S1*	11	rs1483121	(92)	EA	G/A	Downstream	0.86	0.006 (0.008)	0.034	FG_BMI30
*P2RX2*	12	rs10747083	(22)	EA	A/G	Upstream	0.66	0.013 (0.002)	7.60 × 10^−9^	FG
*PAM*	5	rs35658696	(38)	EA	G/A	D563G	0.05	−0.152 (0.027)	1.9 × 10^−8^	Insulinogenic index
*PCSK1*^b^	5	rs13179048	(92)	EA	C/A	Downstream	0.69	0.027 (0.013)	0.022	FG_BMI30
		rs4869272	(22)	EA	T/C	Downstream	0.69	0.018 (0.002)	1.00 × 10^−15^	FG
		rs6234	(40)	EA	C/G	Q665E	0.28	−0.022 (-0.004)	3.00 × 10^−8^	FG_adjBMI
		rs6235	(40)	EA	G/C	S690T	0.28	−0.022 (-0.004)	4.10 × 10^−8^	FG_adjBMI
		rs6235	(24)	EA	G/C	S690T	0.28	0.039 (0.005)	9.8 × 10^−27^	Proinsulin
*PDGFC*	4	rs4691380	(92)	EA	C/T	Intronic	0.67	0.020 (0.010)	0.072	FI_BMI30
		rs6822892	(22)	EA	A/G	Intronic	0.68	−0.003 (0.019)	4.00 × 10^−8^	HOMA-IR
								0.014 (0.002)	2.60 × 10^−10^	FI_adjBMI
*PDK1/RAPGEF4*	2	rs733331	(48)	EAA	A/G	Intronic	0.56	0.036 (0.006)	6.98 × 10^−11^	FG_adjBMI
*PDX1*^b^	13	rs2293941	(92)	EA	A/G	Upstream	0.22	0.016 (0.006)	0.0078	FG_BMI30
		rs11619319	(22)	EA	G/A	Upstream	0.23	0.019 (0.002)	1.30 × 10^−15^	FG
*PELO*	5	rs6450057	(46)	EA	T/C	Intergenic	0.37	−0.011 (NR)	9.21 × 10^−5^	FI_adjBMI
				AA	T/C	Intergenic	0.40	0.027 (NR)	3.11 × 10^−6^	FI_adjBMI
*PEPD*	19	rs731839	(22)	EA	G/A	Intronic	0.34	0.015 (0.003)	1.70 × 10^−8^	FI
								0.015 (0.002)	5.10 × 10^−12^	FI_adjBMI
*PPARG*^b^	3	rs17036328	(22)	EA	T/C	Intronic	0.86	0.021 (0.003)	3.60 × 10^−12^	FI_adjBMI
*PPIP5K2*	5	rs36046591	(38)	EA	G/A	S1228G	0.05	−0.152 (0.027)	2.3 × 10^−8^	Insulinogenic index
*PPP1R3B*	8	rs4841132	(92)	EA	A/G	Upstream	0.10	0.054 (0.021)	0.0031	FG_BMI30
		rs983309	(22)	EA	T/G	Upstream	0.12	0.032 (0.016)	0.00073	FI_BMI30
		rs2126259	(22)	EA	T/C	Upstream	0.11	−0.055 (0.028)	2.00 × 10^−8^	HOMA-IR
		rs11782386	(22)	EA	C/T	Upstream	0.87	0.026 (0.003)	6.30 × 10^−15^	FG
								0.029 (0.004)	3.80 × 10^−14^	FI
								0.099 (0.017)	2.20 × 10^−9^	2hG
								0.024 (0.003)	3.30 × 10^−13^	FI_adjBMI
*PROX1*	1	rs340874	(80)	EA	C/T	Upstream	0.52	0.013 (0.003)	6.60 × 10^−6^	FG
*RMST*	12	rs17331697	(93)	EA	T/C	Intronic	0.90	0.046 (0.007)	1.30 × 10^−11^	FG
*RREB1*^b^	6	rs17762454	(22)	EA	T/C	Intronic	0.26	0.014 (0.002)	9.60 × 10^−9^	FG_adjBMI
*RSPO3*	6	rs2745353	(22)	EA	T/C	Intronic	0.51	0.014 (0.002)	5.50 × 10^−9^	FI
*SC4MOL*	4	rs17046216	(45)	AA + West African	A/NR	Intergenic	NR	0.180 (0.030)	1.65 × 10^−8^	FI_adjBMI
								0.190 (0.030)	2.88 × 10^−8^	HOMA-IR
*SGSM2*	17	rs4790333	(24)	EA	T/C	Intronic	0.45	0.015 (0.004)	3.00 × 10^−9^	Proinsulin
		rs61741902	(38)	EA	A/G	V996I	0.01	0.126 (0.021)^a^	8.70 × 10^−10^	Fasting proinsulin
*SIX2*/*SIX3*	2	rs895636	(47)	EAA	T/C	Intergenic	0.38	0.039 (0.006)	9.99 × 10^−13^	FG
*SLC2A2*^b^	3	rs11920090	(80)	EA	T/A	Intronic	0.87	0.02 (0.004)	3.30 × 10^−6^	FG
*SLC30A8*^b^	8	rs13266634	(80)	EA	C/T	R325W	NR	0.027 (0.004)	5.50 × 10^−10^	FG
		rs11558471	(94)	EA	C/T	3’UTR	0.70	0.02 (NR)	5.00 × 10^−8^	HbA1c
			(22)	EA	A/G		0.68	0.029 (0.002)	7.80 × 10^−37^	FG
			(24)	EA	A/G		0.69	0.028 (0.005)	3.1 × 10^−18^	Proinsulin
*SNX7*	1	rs9727115	(24)	EA	G/A	Intronic	0.64	0.013 (0.005)	2.40 × 10^−10^	Proinsulin^d^
*SPTA1*	1	rs2779116	(90)	EA	T/C	Intronic	0.28	0.024 (0.004)	2.75 × 10^−9^	HbA1c
*TBC1D30*	12	rs150781447	(38)	EA	T/C	R279C	0.02	0.204 (0.025)	1.3 × 10^−16^	Proinsulin AUC_30-120_
*TCERG1L*	10	rs7077836	(45)	AA + West African	T/NR	Intergenic	NR	0.280 (0.050)	7.50 × 10^−9^	FI_adjBMI
								0.340 (0.050)	4.86 × 10^−20^	HOMA-IR
*TCF7L2*	10	rs7903146	(22)	EA	T/C	Intronic	0.28	0.022 (0.002)	2.70 × 10^−20^	FG
		rs12243326	(22)	EA	C/T	Intronic	0.28	−0.018 (0.003)	6.10 × 10^−11^	FI
			(24)	EA			0.30	0.032 (0.007)	2.3 × 10^−20^	Proinsulin
			(95)	EA			0.28	0.05 (0.03)	1.48 × 10^−7^	HbA1c
			(89)	EA			0.21	0.07 (0.01)	4.23 × 10^−10^	2hG_adjBMI
*TET2*	4	rs9884482	(22)	EA	C/T	Intronic	0.35	0.017 (0.002)	1.40 × 10^−11^	FI
		rs974801	(22)	EA	G/A	Intronic	0.39	0.014 (0.002)	3.30 × 10^−11^	FI_adjBMI
*TMEM79*	1	rs6684514	(49)	EAA	G/A	V147M	0.76	0.09 (0.01)	1.30 × 10^−23^	HbA1c
*TMPRSS6*	22	rs855791	(90)	EA	A/G	V736A	0.42	0.027 (0.004)	2.74 × 10^−14^	HbA1c
*TOP1*/*ZHX3*^b^	20	rs6072275	(22)	EA	A/G	Intronic	0.16	0.016 (0.003)	1.70 × 10^−8^	FG
*UHRF1BP1*	6	rs4646949	(92)	EA	T/G	Intronic	0.75	0.009 (0.010)	0.16	FI_BMI30
		rs6912327	(22)	EA	T/C	Intronic	0.80	0.017 (0.003)	2.30 × 10^−8^	FI_adjBMI
*URB2*	1	rs141203811	(40)	EA	T/A	E594V	0.001	0.282 (-0.066)	3.10 × 10^−7^	FI_adjBMI
*VPS13C/C2CD4A/B*	15	rs17271305	(89)	EA	G/A	Intronic	0.42	0.060 (0.010)	4.11 × 10^−8^	2hG_adjBMI
		rs11071657	(80)	EA	A/G	Downstream	0.63	0.008 (0.003)	0.01	FG
		rs4502156	(24)	EA	T/C	Downstream	0.58	0.029 (0.004)	3.5 × 10^−20^	Proinsulin
*WARS*	14	rs3783347	(22)	EA	G/T	Intronic	0.79	0.017 (0.003)	1.30 × 10^−10^	FG
*YSK4*	2	rs1530559	(22)	EA	A/G	Intronic	0.52	0.015 (0.003)	3.40 × 10^−8^	FI
*ZBED3*	5	rs7708285	(22)	EA	G/A	Intronic	0.27	0.015 (0.003)	1.20 × 10^−8^	FG_adjBMI

Chr, Chromosome; EA, European ancestry; EAA, East Asian ancestry; AA, African American ancestry; Allele, [E, Effect allele/O, Other allele]; EAF, Effect allele frequency; NR, Not reported/available; FG, fasting glucose (mmol/L); FG_adjBMI, fasting glucose BMI adjusted; FG_BMI30, Fasting glucose in individuals with BMI = 30 kg/m^2^; FI, fasting insulin (pmol/L); FI_adjBMI, fasting insulin BMI adjusted; 2hG, 2 h glucose (mmol/L); HbA1c, glycated haemoglobin (%); HOMA-B, β-cell function by homeostasis model assessment; HOMA-IR, insulin resistance by homeostasis model assessment; Proinsulin (pmol/L); ISI_adjBMI, Modified Stumvoll Insulin Sensitivity Index, adjusted for BMI. Effect estimates are taken from original references are all rounded to three decimal points.

Effect sizes for ISI are presented as the SD per effect allele.

a Coefficient units are ln(pmol/l).

b Likely effector transcript at the locus.

c Signal at NAT2 did not reach genome-wide significance.

d Signal at SNX7 reached genome-wide significance after adjusting for fasting glucose (*P = *5.4 × 10^−9^).

## Common Variant Trait Associations

Genome-wide association studies (GWAS) have transformed the landscape of glycaemic trait genetics. Prior to GWAS FG was associated with genetic variants in *GCK* (Glucokinase) (15). Subsequently, early GWAS replicated the *GCK* association (16,17) and identified novel associations with FG at *G6PC2* (16,17) and *GCKR* (18–20). Aggregation of data through meta-analyses, primarily in populations of European ancestry in the setting of large consortia (such as the Meta-Analyses of Glucose and Insulin-related traits Consortium, MAGIC), and the development of targeted arrays such as the Metabochip (21), have increased the number of associations between common variants and the most commonly used glycaemic measures (FG, FI, 2hG and HbA1c) to over 70 (Table 1), accounting for <6% of phenotypic variance in Europeans (22,23).

Association with more sophisticated glycaemic measures, identified additional genome-wide significant loci, such as *LARP6* and *SGSM2* associated with fasting proinsulin (24), *NAT2* associated with euglycemic clamp and insulin suppression test techniques (25), *BCL2* and *FAM19A2* associated with the modified Stumvoll Insulin Sensitivity Index (ISI) (a dynamic measure of whole-body insulin sensitivity) (26). These measures enabled detailed physiological characterization of existing loci (27–29), including establishment of the role of *MTNR1B* in decreased early phase insulin response (30). An alternative measure of impaired glucose tolerance, 1-h glucose (1hG), may warrant further research following studies investigating its potential utility (31,32), and the identification of novel loci *MYL2*, *C12orf51* and *OAS1* associated 1hG in Koreans (33) (Table 1).

## The Contribution of Low Frequency and Rare Variants

The majority of genome-wide association signals are both common and non-coding, and recent efforts have focused on the contribution of rare (MAF < 1%) and low frequency (1% ≤ MAF < 5%) variants, and their role as possible causal variants. Current strategies include: 1) genotyping arrays targeting the exons (also known as ‘Exome Chips’) or with combined common variant backbone and exonic content; 2) genome- and exome –wide sequencing and 3) combined genotyping arrays and dense imputation using sequence based reference panels such as 1000 genomes (34), UK10K (35,36) and HRC (37).

Huyghe *et al.* (38) were the first to demonstrate the utility of exome-array genotyping. Using this approach in Finns, they found novel low-frequency coding variants at *TBC1D30* (R279C, MAF = 2.0%) and *KANK1* (R667H, MAF = 2.9%) associated with fasting proinsulin levels (and late/early-phase proinsulin to insulin conversion ratio, respectively) and two variants with MAF = 5.3%, and in near-perfect LD (*r*^2^=0.997) at *PAM* (D563G) and *PPIP5K2* (S1228G) associated with insulin secretion (insulinogenic index). Novel low frequency variants at previously identified GWAS loci, *SGSM2* (V996I, MAF 1.4%) and *MADD* (R766X, MAF = 3.7%) associated with fasting proinsulin, and common variants associated with insulin secretion or beta-cell function at *GPSM1* (S391L), *HNF1A* (intergenic), and *ABO* (intronic) were also identified. Gene-based tests (aggregating rare/low frequency variants at the locus) identified significant associations with fasting proinsulin at *TBC1D30, SGSM2* and *ATG13*, although conditional analyses suggested the *ATG13* signal was partially driven by variants in *MADD.* Wessel *et al.* (39) identified a non-synonymous variant at *GLP1R* (A316T; rs10305492; MAF = 1.4%) associated with lower FG, early insulin secretion and type 2 diabetes risk, but higher 2hG (39). The same effort identified a gene-based signal at *G6PC2*, which was driven by three non-synonymous rare variants (H177Y, Y207S and S324P) and a stop variant (R283X). Further evidence of FG association at *G6PC2* was provided by Mahajan *et al.* (40), who also found multiple rare coding variants at this gene (V219L, H177Y, Y207S), with evidence of loss of protein function, identifying *G6PC2* as an effector transcript at the *G6PC2/ABCB11* locus (Table 1). The same study identified 10 additional non-synonymous coding variants associated with FG or FI, of which eight mapped to known GWAS loci: *GCKR* (P446L), *SLC30A8* (R325W), *RREB1* (S1554Y), *PCSK1* (S690T, Q665E), *COBLL1* (N939D), *TOP1* (N310S) and *PPARG* (P12A) (Table 1). Two novel loci, *GLP1R* [A316T, supporting result from (39)] and *URB2* (E594V) were also identified. Despite this success only two association signals were low frequency variants, H177Y MAF 0.8% at *G6PC2/ABCB11* and E594V MAF 0.1% at *URB2*, (Table 1), and the data supported *PCSK1*, *RREB1* and *ZHX3* as likely effector transcripts at the associated loci, with *RREB1* also replicated in a type 2 diabetes study (7), confirming it as the probable effector gene for T2D at the *SSR1* locus.

The UK10K Consortium (35) performed low depth (7x) whole-genome sequencing in 3,781 participants from two British cohorts (ALSPAC and TwinsUK) and conducted association analyses with 31 phenotypes available in both cohorts, replicating common variant associations at *G6PC2-ABCB11* with FG. Subsequent fine-mapping efforts identified missense variant associations as the causal variant or within the credible set of causal variants at *GCKR* (L446P) and *SLC30A8* (R325W) (41).

## Transferability to Other Ancestries and Fine Mapping

Driven by the availability of large sample sizes, the majority of early GWAS studies were performed in populations of European ancestry. Since then, efforts have expanded to diverse populations, leveraging differences in allele frequency and linkage disequilibrium (LD) structure, to harness power for novel locus discovery and fine-mapping (42). While genetic effect sizes for common variants are largely consistent across ancestry groups, allele frequencies can vary (43,44), improving power for association in certain populations.

Studies in African Americans have identified *SC4MOL* and *TCERG1L* associated with FI and insulin resistance (HOMA-IR) (45), and *FAM133A* and *PELO* associated with FI, where *PELO* was identified in a trans-ethnic meta-analysis combining African American data with publicly available European summary statistics from MAGIC (46). In East Asians, studies have identified *SIX2-SIX3*, *C12orf51*, *PDK1-RAPGEF4*, *KANK1* and *IGF1R* associated with FG (33,47,48), *MYL2*, *C12orf51* and *OAS1* associated with 1-2hG (33) and *HBS1L-MYB*, *CYBA*, *MYO9B* and *G6PC3* for HbA1c (49,50) (Table 1).

More focused replication and fine-mapping efforts have also been carried out in African Americans (51–53), Asian populations (54,55) and an admixed Mexican population (56). Exact (the same index variant) and local replication has replicated variants in or near *MNTR1B*, *G6PC2-ABCB11*, *GCK*, *IRS1*, *TCF7L2*, *DGKB*, *FADS1*, *GCKR*, *SLC30A8* and *ZMAT4* associated with FG and *GCKR* with FI. These results suggest partial locus transferability but are limited in power by the relatively modest sample sizes (largest discovery sample sizes, N∼20-25 K) compared to the much larger European ancestry efforts (N∼ 108-133 K for FI and FG) that have led to the discovery of the loci being assessed. Nonetheless they highlight the utility of diverse populations to refine association signals, to fewer probable casual variants. For example, inclusion of African American samples in a trans-ethnic fine-mapping approach reduced the credible set (smallest set of SNPs that accounts for 99% of the posterior probability of containing the causal variant at the locus) at *GCK* and *ADCY5* for FG, *PPP1R3B* for FI, and *GCKR* for FG and FI, to a single SNP (46).

In contrast, population isolates derive from a small number of founder individuals, have reduced genetic diversity and higher levels of LD, and enrichment of some rare alleles following the initial bottleneck, thus increasing power and facilitating genetic discovery (57,58). Successful outcomes are the *TBC1D4* locus identified in Greenland strongly associated with 2hG and 2hI (59), and most recently, a variant (P50T) in *AKT2* associated with a large effect (12% increase) on FI, with MAF 1.1% in Finns, but virtually absent (MAF ≤0.2%) in the individuals from other ancestries (60).

## Biological and Functional Insights

As mentioned earlier, most glycaemic trait genetic variant associations map within non-coding regions, with the underlying causal or effector transcript hard to establish, requiring fine-mapping which often necessitates other genomic evidence to establish a functional link between associated variants and underlying biology. Recent studies have shown that pancreatic islet enhancers are enriched with FG associated loci (61,62), and that pancreatic islet eQTLs provide important clues for candidate effector transcripts at FG associated loci (63,64). For some of these loci, the eQTL provides compelling confirmatory evidence for the biological candidate loci at these association signals [*e.g. ADCY5*, *DGKB* at the *DGKB/TMEM195* locus, *FADS1* and *MTNR1B* (63), replicating previous findings at this locus (64,65)]. At the *ARAP1* locus a recent study (63) suggests *STARD10* is the likely effector transcript, which is in contrast with earlier data (66), but consistent with another more recent report (67). At the *MADD* locus two potential effector transcripts were identified, *MADD* and *ACP2* (63), supporting evidence for *MADD* is provided by a beta-cell specific mouse model which showed that *Madd* plays a role in glucose-stimulated insulin secretion (68), however the mouse phenotype did not provide any clues regarding the insulin processing effects also strongly associated with MADD (24). *ACP2*, on the other hand, encodes a lysosomal protein; the role of lysomes in the degradation of ageing insulin granules (69) was hypothesised by the authors (63) as a possible link for the fasting glucose and prosinsulin association signals. *WARS*, *NKX6-3* (at the *ANK1* locus) and *RBMA6* (at the *AMT* locus) were also implicated as plausible effector transcripts but the mechanism through which they impact islet function, is as yet, unknown (63).

Loci associated with insulin resistance have been more recalcitrant to the GWAS approach and thus the number of established loci and effector transcripts is much smaller (Table 1). Recently, a blood transcriptomic genome-wide analysis (TWAS) combined with eQTL analysis, identified a trans-eQTL (rs592423) where the A-allele was associated with higher *IGF2BP2* transcript levels and higher fasting insulin, suggesting this is the effector transcript at this locus (70). The TWAS also identified several genes with established roles in metabolic traits, namely *IRS2* and *FOXO4* involved in insulin signalling, and three genes involved in adipocyte or adipokine biology (*ITLN1, PID1, ADIPOR1*) (70). Another recent approach focused on identifying loci simultaneously associated with higher levels of FI adjusted for BMI, higher levels of triglycerides and lower levels of HDL, a hallmark of insulin resistance and of the condition lipodystrophy. In total, 53 associated loci were identified which when combined in a genetic risk score, were associated with increased T2D and coronary heart disease risk, but lower peripheral adipose tissue. The same loci also provided the first evidence of polygenic influence in familial lipodystrophy type 1, a severe form of insulin resistance previously thought to be monogenic in origin. Overall, these data suggested that impaired peripheral adipose tissue capacity may be an important mechanism influencing insulin resistance and is likely to be an important aetiological contributor to insulin-resistant cardiometabolic disease (13). The importance of adipose tissue differentiation in insulin resistant states was known from monogenic lipodystrophy due to mutations in *PPARG* (71,72) and has also more recently been demonstrated to be an important aetiological factor in T2D predisposition (73).

Complementing functional regulatory associations, the identification of multiple rare missense variants shown to affect protein function, and that contribute to a gene-based association signal, is a strong indicator that the effector transcript has been identified [*e.g. G6PC2* (39,40), *SLC30A8* (74) and *PPARG* (73)]. Similarly, single-point associations shown, or predicted, to have an effect on protein function [*e.g.* the P50T variant at *AKT2* associated with FI (60) and the S690T and Q665E at *PCSK1* associated with proinsulin and FG (24,40)], or mapping proximal to classical candidate loci are also strong indicators that the effector transcript is likely to map to those specific genes. This approach suggested that *SLC2A2* (encoding GLUT2), *GCK*, *GCKR, FOXA2* and *PDX1* are the likely effector transcripts at these loci (Table 1). *SLC2A2* encodes GLUT2, the main glucose transporter in the islets of rodents but not of humans, where GLUT1 and GLUT3 predominate both in islets and beta-cells, suggesting that the role of variants at this gene are likely to be mediated through effects on other metabolic tissues (75). Recently, another study has supported this hypothesis, where the C allele of rs8192675 in *SLC2A2* was associated with a greater metformin-induced decrease in HbA1c levels, and was also shown to be an eQTL for GLUT2 in human liver samples. This suggested a role of hepatic GLUT2 in metformin action and glucose metabolism with significant clinical impact, and proposed as a biomarker for precision medicine (76). The importance of the liver in glucose homeostasis and FG levels, was also confirmed by studies of the P446L variant in *GCKR*, which demonstrated that this variant affected *GCKR* inhibition of *GCK* which was predicted to promote hepatic glucose metabolism with consequent decrease in FG (77). A number of glycaemic trait-associated loci map within, or proximal to, genes associated with a range of Mendelian metabolic disorders namely *SLC2A2* (OMIM # 227810), *GCK* (OMIM # 125851), *PPARG* (OMIM # 604367), *PCSK1* (OMIM # 600955), *PDX1* (OMIM # 606392), *GLIS3* (OMIM # 610199), *IGF1* (OMIM # 608747) and *HNF1A* (OMIM # 600496) providing additional biological support for their candidacy as effector transcripts at these loci, and suggesting a role for rare penetrant and common variants influencing familial or polygenic traits, respectively.

These data combined, highlight genes involved in glucose regulation, insulin processing, secretion and response, and transcription factors with an established role in pancreas development as important mechanisms influencing glycaemic traits. Early GWAS results highlighted for the first time in humans, the role of loci involved in circadian rhythm [*MTNR1B* (65,78,79) and *CRY2* (80)] in glucose metabolism. These results have been replicated in many additional studies, and subsequent analyses have shown that the associations at these loci are season-dependent (81) and that clock genes are regulated in pancreatic islet cells confirming that perturbations in circadian clock components are likely important in glucose homeostasis (82). The role of circadian clock in metabolism and possible therapeutic opportunities has recently been extensively reviewed (83), though the exact mechanism of how *MTNR1B* is likely to affect glucose homeostasis and diabetes risk remains the subject of some controversy (84,85).

## Glycaemic Traits and T2D

Fasting glucose is used to diagnosis type 2 diabetes (T2D) however, GWAS studies have demonstrated that the genetic architecture of these two traits does not fully overlap (22,80,86), suggesting that raising fasting glucose *per se* is insufficient to confer T2D risk and that pathophysiology is likely conditional on the affected pathway. The availability of detailed measures of glycaemia has thus helped demonstrate that a diverse set of mechanisms are involved in conferring risk of T2D. To date, T2D risk loci have been grouped into five distinct groups: a) those loci whose primary effect appears to be on insulin sensitivity (*PPARG, KLF14, IRS1, GCKR*); b) loci associated with decreased insulin secretion and with fasting hyperglycaemia (*MTNR1B, GCK*); c) a single locus, *ARAP1*, associated with impaired proinsulin processing; d) a large cluster of loci influencing insulin processing and secretion with modest or no detected effects on fasting glucose levels (*TCF7L2, SLC30A8, HHEX/IDE, CDKAL1, CDKN2A/2B, PROX1, THADA, ADCY5, DGKB/TMEM195*); and e) a large set of 20 loci that despite influencing T2D risk did not have clear associations with any of the available measures of glycaemia and which may correspond to novel mechanisms influencing diabetes by as yet not understood biology (87). Similar earlier analyses of loci influencing fasting and post-challenge glucose measures also suggested similar diverse mechanisms influencing these traits (27).

A recent large-scale trans-ethnic meta-analyses of GWAS for HbA1c has expanded the number of HbA1c-associated loci to 60, and importantly highlighted that the genetic architecture of the trait differed in African Americans compared to the other ancestries studied (European, East and South Asians). In African Americans, a single variant in the *G6PD* gene (G202A) responsible for glucose-6-phosphate deficiency, accounted for a significant fraction of the variance in the trait (14.4%) and led to a substantial decrease in HbA1c values in hemizygous men (0.81%-units) and homozygous women (0.68%-units). This variant, if unaccounted for, could lead to up to 2% of African Americans with T2D to remain undiagnosed, highlighting the importance of studying glycaemic traits in diverse populations in order to avoid racial health disparities in the application of precision medicine (23).

## Summary and Future Directions

In conclusion, large-scale genetic association analyses, combined with information on genomic features (enhancers, expression QTLs, TWAS) and high- throughput functional assays (88) have provided an increasingly growing list of loci associated with continuous glycaemic measures. The genetic architecture of these traits is comprised of many common variants of modest effect, mostly mapping to non-coding regions, with evidence of enrichment in active islet enhancers, and some overlap with monogenic loci involved in various disorders of metabolism. Genetic locus overlap between several glycaemic traits can be observed, most notably between FG and many of the other glycaemic traits, including T2D, though this number is likely to change as larger more powered studies become available (Fig. 1). Interestingly, FG and FI, have limited overlap in associated loci which may be a reflection of underlying differences in physiology affecting these traits (Fig. 1). These approaches have revealed some expected, and some novel pathways involved in glucose homeostasis, with recent efforts highlighting a number of low-frequency or rare missense variants affecting protein function, which provide compelling evidence for the effector transcript at a given locus. Studies of diverse populations have demonstrated, for the most part, the transferability of glycaemic trait-associated loci across ancestries and highlighted the power of isolated populations to identify variants of larger effect sizes. More recently, large-scale trans-ethnic genetic analysis of HbA1c highlighted the need for more powered studies on diverse ancestries to avoid health disparities in the application of genomics to the clinic. Future efforts combining sequencing approaches, increased sample sizes (particularly in non-European ancestries), understanding of the non-coding regions of the genome and the integration of other ‘omics’ data will continue to improve understanding of the biology underlying glycaemic traits and how they impact on disease.
